# Membrane adaptation limitations in *Enterococcus faecalis* underlie sensitivity and the inability to develop significant resistance to conjugated oligoelectrolytes

**DOI:** 10.1039/c7ra11823f

**Published:** 2018-03-13

**Authors:** Gayatri Shankar Chilambi, Iris H. Gao, Bo Kyeong Yoon, Soohyun Park, Lisa M. Kawakami, Vikashini Ravikumar, Mary B. Chan-Park, Nam-Joon Cho, Guillermo C. Bazan, Kimberly A. Kline, Scott A. Rice, Jamie Hinks

**Affiliations:** Interdisciplinary Graduate School, Nanyang Technological University Singapore 637141; Singapore Centre for Environmental Life Sciences Engineering (SCELSE), Nanyang Technological University Singapore 637551 jhinks@ntu.edu.sg rscott@ntu.edu.sg; Weill Cornell Graduate School of Medicine 1300 York Ave New York NY USA 10065; School of Materials Science and Engineering, Nanyang Technological University 50 Nanyang Avenue Singapore 639798; School of Chemical and Biomedical Engineering, Nanyang Technological University Singapore 637459; Department of Chemistry & Biochemistry and Materials, Center for Polymers and Organic, Solids, University of California Santa Barbara California USA 93106; School of Biological Sciences, Nanyang Technological University Singapore 637551; The ithree Institute, The University of Technology Sydney Sydney New South Wales Australia; Centre for Biomimetic Sensor Science, Nanyang Technological University Singapore

## Abstract

The growing problem of antibiotic resistant bacteria, along with a dearth of new antibiotics, has redirected attention to the search for alternative antimicrobial agents. Conjugated oligoelectrolytes (COEs) are an emerging class of antimicrobial agents which insert into bacterial cell membranes and are inhibitory against a range of Gram-positive and Gram-negative bacteria. In this study, the extent of COE resistance that *Enterococcus faecalis* could achieve was studied. *Enterococci* are able to grow in hostile environments and develop resistance to membrane targeting antibiotics such as daptomycin in clinical settings. Herein we expand our knowledge of the antimicrobial mechanism of action of COEs by developing COE-resistant strains of *E. faecalis* OG1RF. Evolution studies yielded strains with a moderate 4–16 fold increase in antimicrobial resistance relative to the wild type. The resistant isolates accumulated agent-specific mutations associated with the *liaFSR* operon, which is a cell envelope-associated stress-response sensing and regulating system. The COE resistant isolates displayed significantly altered membrane fatty acid composition. Subsequent, exogenous supplementation with single fatty acids, which were chosen based on those dominating the fatty acid profiles of the mutants, increased resistance of the wild-type *E. faecalis* to COEs. In combination, genetic, fatty acid, and uptake studies support the hypothesis that COEs function through insertion into and disruption of membranes and that the mechanism by which this occurs is specific to the disrupting agent. These results were validated by a series of biophysical experiments showing the tendency of COEs to accumulate in and perturb adapted membrane extracts. Collectively, the data support that COEs are promising antimicrobial agents for targeting *E. faecalis*, and that there is a high barrier to the emergence of severely resistant strains constrained by biological limits of membrane remodeling that can occur in *E. faecalis*.

## Abstract 2

COEs are emerging antimicrobials to combat drug resistant infections and to which bacteria develop only limited resistance.

## Graphical abstract

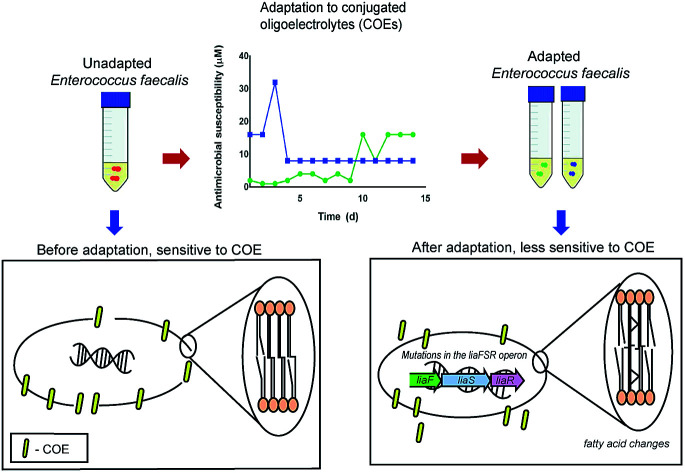

## Introduction

Antimicrobial resistance (AMR) is of increasing global concern. To stave off an impending and much feared post-antibiotic era, a high level discussion was convened by the UN General Assembly in September 2016.^1^ The meeting declaration addressed the need to improve the investment in R&D to foster the development of alternative antibiotics. In addition the regulation of antibiotic usage in animal husbandry and agriculture along with limitations on their sale are among other measures recommended to combat the challenge of AMR at a global level. Antibiotic discovery was prolific between 1940 and 1960 during which time all of the major classes of antibiotics in clinical use today were discovered. Even with the application of innovative approaches to antibiotic discovery, including target-based, high-throughput methods and the mass screening of novel metabolites from uncultured microorganisms, no new classes of antibiotics were introduced until after 2000, more than four decades after the introduction of quinolones in 1962.^2,3^ All recently approved antimicrobial drugs belong to existing classes of antibiotics such as the glycopeptides (dalbavancin (2014), oritavancin (2014) and televancin (2013)) or cephalosporins (ceftaroline (2010)).^4,5^ Unfortunately, antimicrobial discovery has not kept pace with microbial adaptation and AMR has been described for all of the existing classes of antibiotics.^5^

Many promising antimicrobial compounds that are in the early stage of development, such as the recently discovered malacidin from genetic mining (targets lipid II),^6^ teixobactin from soil (targets lipid II and lipid III),^2^ sortase transpeptidase inhibitors (targets sortases),^7^ lipoteichoic acid inhibitors,^8^ antimicrobial peptides (AMPs),^9^ and nisin-derived lipopeptides,^10^ all target the cell envelope to some degree, although further research is needed to ensure their clinical utility.^11,12^ Based on assumptions that the 20 different antibiotic classes introduced between 1930–1962 were relevant for 50 years,^5^ Coates *et al.* (2011), suggest that at least 20 new classes of antibiotics are required to be developed between 2000–2050 in order to cope with the growing threat of AMR. This highlights the urgent need to identify new classes of antimicrobials with activity against drug-resistant strains or those which do not elicit AMR.

Conjugated oligoelectrolytes (COE) are a class of compounds defined by phenylene-vinylene repeat units with ionic-pendant groups at the molecular termini. Because their charge distribution and hydrophobicity mimics that of phospholipids, and perhaps because of their ability to self-assemble, these amphiphiles have an affinity for microbial membranes.^13,14^ They have been applied in the development of solar cells, nanoarchitectonics and more recently in modifying bacterial membranes.^15^

**Fig. 1:**
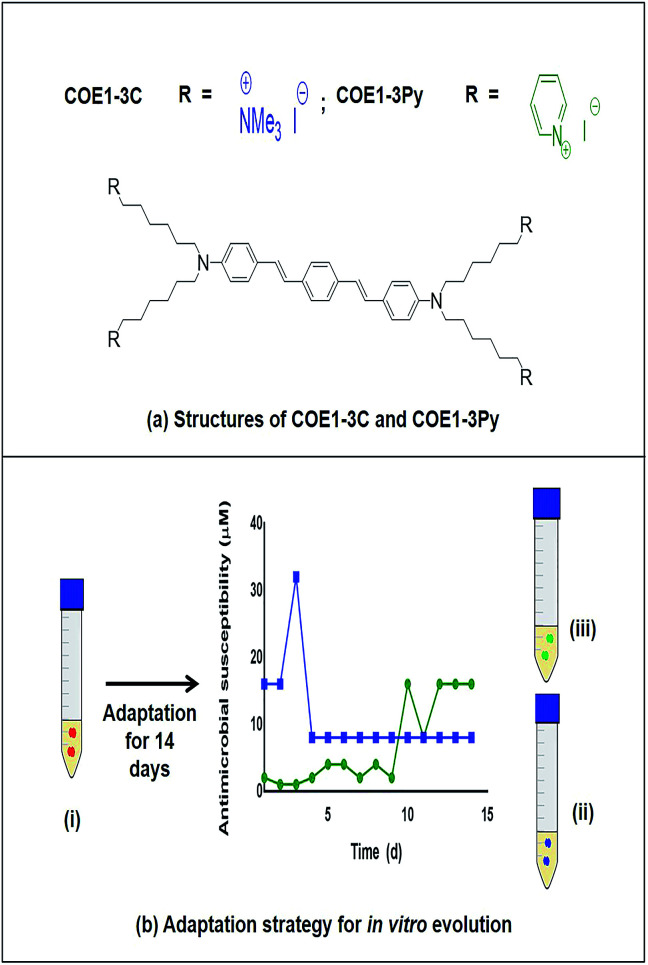
(a) Structures of COE1-3C and COE1-3Py (b) adaptation strategy for *in vitro* evolution – (i) *Enterococcus faecalis* OG1RF wild type, (ii) COE1-3C adapted strain and (iii) COE1-3Py adapted strain were used for the characterization studies indicated. Blue and green lines indicate evolution in COE1-3C and COE1-3Py. These strains were subjected to *MIC tests*, *whole genome sequencing* to identify mutations associated with the adaptations, *fatty acid profile analysis* and lastly, *lipid extraction* to study the *biophysical interaction* of COEs with lipid bilayers using quartz crystal microbalance-dissipation (QCM-D) and electrochemical impedance spectroscopy (EIS).

The antimicrobial function of COEs has been related to specific structural features and the most potent antimicrobial structures among two related classes of COE have a backbone of three aromatic repeat units differing only in the terminal charge moiety with COE1-3C (1,4-bis(4′′-(*N*,*N*-bis(6′′′′-(*N*,*N*,*N*-trimethylammonium)hexyl)amino)-styryl)benzene tetraiodide) being terminated by a tetraalkylammonium group and COE1-3Py ((1,4-bis(4′′-(*N*,*N*-bis(6′′′′-(pyridinium)hexyl)amino)-styryl)benzene tetraiodide)) with a pyridinium ion (Fig. 1).^16^ Although the antimicrobial potency of these compounds against bacteria has been demonstrated, and the mode of action is hypothesized to be through the membrane disrupting properties of COEs, little is known about the mechanism of action.^16,17^ Additionally, resistance to this new class of antimicrobials has not been explored to date. Such information is vital since a molecular level understanding of COE interactions with membranes is necessary to direct synthetic design towards the discovery of more potent analogues.

Here, we have explored the effect of COE1-3C and COE1-3Py on *Enterococcus faecalis* cell membranes. In particular, resistance to COE1-3C and COE1-3Py was developed and the accompanying fatty acid changes were surveyed to shed light on natural adaptation to COEs. We have applied standard minimum inhibitory concentration (MIC) tests to look at cross-resistance with other membrane disrupting antibiotics as well as drawing on biophysical assays with model membranes to assess lipid composition-dependent membrane binding and the resulting membrane perturbations caused by these compounds. Whole genome sequencing of resistant isolates identified mutations in genes associated with the regulation of cell envelope stress responses in *E. faecalis* OG1RF, suggesting that the primary mechanism of resistance is through membrane reorganization, presumably to modulate uptake, ordering, and packing parameters to counter COE-mediated membrane perturbations.

## Materials and methods

### Conjugated oligoelectrolytes

COE1-3C and COE1-3Py were synthesized as described by Garner *et al.*, (2010) and Yan *et al.*, (2016), respectively.

### Determination of MIC

MIC tests were conducted using broth microdilution methods based on a method reported by Wiegand *et al.* (2008). Experiments were carried out in triplicate and compounds were serially diluted following a log_2_ series in Brain Heart Infusion (BHI) broth. Growth in each of the wells was assessed after 18 h incubation at 37 °C. MIC was defined here as the lowest concentration of antibiotic that inhibited visible growth as observed with the unaided eye.^18^

### Development of *in vitro* resistance to COEs in *E. faecalis* OG1RF

COE1-3C and COE1-3Py resistant strains of *E. faecalis* OG1RF were generated by serial passage in BHI medium containing increasing concentrations of either COE1-3C or COE1-3Py in triplicate. On day 1, the optical density (OD_600_) of *E. faecalis* OG1RF was adjusted to 0.1, then cultures were exposed daily to two fold increase in COE concentrations beginning at 0.5× MIC. On a given day, the culture with the highest concentration of COE that showed visible growth of *E. faecalis* OG1RF after 24 h incubation was used as the inoculum for the subsequent day and incubated at both the concentration of COE from which it was taken and at a twofold increment above that concentration. Serial passaging was continued over 14 d to ensure further resistance did not occur. Daily aliquots were recovered from the serial passage cultures for each of the experiments and stored at −80 °C. Each isolate collected from these experiments was passaged 3–4 times in BHI medium free of COE and the MIC of the tolerant strains was once again determined to ensure that the resistant phenotype was maintained. The strains that were resistant to COE1-3C and COE1-3Py were designated EFC3C and EFC3Py respectively.

### Cross resistance between EFC3C and EFC3Py

Cross resistance between EFC3C and EFC3Py strains to COE1-3C and COE1-3Py was determined from the MIC using broth microdilution tests as described above. In addition, cross resistance to daptomycin (A.G. Scientific Inc.) was examined as it is an antimicrobial agent whose mechanism of action can be reasonably assumed to be similar to COEs.^16,17^

### Whole genome sequencing of EFC3C and EFC3Py

Genomic DNA was harvested from overnight cultures of EFC3C and EFC3Py in BHI broth using an Invitrogen Pure Link DNA Mini Kit. DNA was sequenced on an Illumina MiSeq (Illumina, CA, USA) with 300 bp paired-end sequences and sequences were assembled using CLC Genomics Workbench 8.0. The sequenced genomes were mapped and annotated against the *E. faecalis* OG1RF reference genome (NC_017316) from the NCBI database. The threshold variant frequency was set at 35%. Synonymous mutations were removed for further analysis and structural variations (such as deletions, insertions, inversions and translocation) were confirmed manually on the mapping track as previously described.^21^

### Fatty acid methyl ester (FAME) analysis

Five replicates of *E. faecalis* OG1RF wild type, EFC3C and EFC3Py were grown for 12 h in BHI at 37 °C. Overnight suspensions were centrifuged at 9000 *g* for 2 min and the resultant cell pellets of all three *E. faecalis* strains were freeze dried for 12 h at 80 °C using a Freezone 4.5 plus (Labconco, USA), weighed and then subjected to the Bligh and Dyer method of whole cell lipid extraction. Fatty acid analyses were carried out by the Identification Service of DSMZ, Braunschweig, Germany.

### Effect of fatty acid supplementation on *E. faecalis* OG1RF COE toxicity

*E. faecalis* OG1RF wild type was grown overnight in BHI at 37 °C. The overnight culture was grown to the mid-log phase in BHI with palmitic acid (C_16:0_) (10 μg mL^−1^), *cis*-vaccenic acid (C_18:1 ω7c_) (10 μg mL^−1^) or *cis*-9,10-methyleneoctadecanoic acid (C_19:0 cyclo ω8c_) (12.5 μg mL^−1^), at 37 °C. The fatty acid concentrations were determined based on growth assays with individual fatty acids and solvent controls to ensure there were no inhibitory effects at the test concentration. The conditioned mid-log phase culture was subjected to MIC tests in BHI medium modified with the appropriate fatty acid whose effect on COE sensitivity was of interest.

### Spectroscopic measurement of COE uptake in *E. faecalis* OG1RF

Absorption spectroscopy was used to calculate the percentage of COE uptake in *Escherichia coli* as described in Yan *et al.* (2016). The absorbance of COEs remaining in the solution after *E. faecalis* OG1RF was treated with 5 μM of COE1-3C or COE1-3Py was measured. Cell pellets of *E. faecalis* OG1RF (adjusted to OD 1.0) were washed with phosphate buffer saline (PBS) and resuspended in PBS. The resuspended cells were treated with 5 μM of COE1-3C or COE1-3Py at room temperature. The cells were centrifuged after incubation for 1.5 h and the remaining solution was collected for absorbance scan to quantify the COEs uptake at the wavelength of maximum absorbance. Percentage uptake of 5 μM COE (%) was calculated using the formula: [(*I*_COE_ − *I*_COE+*E. faecalis* S_)/*I*_COE_] × 100. Where *I*_COE_ is the maximum absorbance value of 5 μM of COE1-3C or COE1-3Py; *I*_COE+*E. faecalis* S_ is absorbance value of the supernatant obtained after centrifuging *E. faecalis* OG1RF or EFC3C or EFC3Py (adjusted to OD = 1.0) treated with COEs.

### Extraction of lipids from *E. faecalis* OG1RF, EFC3C and EFC3Py

*E. faecalis* OG1RF wild type, EFC3C and EFC3Py were grown for 12 h in 20 mL of BHI at 37 °C. Overnight suspensions were centrifuged at 9000 *g* for 2 min and the resultant cell pellets of all three *E. faecalis* strains were freeze dried for 12 h at −80 °C using a Freezone 4.5 plus (Labconco, USA), weighed and then subjected to the Bligh and Dyer method of whole cell lipid extraction.^22^ The total lipid extract from the organic phase was dried and resuspended in 1 : 1 (v/v) chilled chloroform: methanol solvent to a final concentration of 10 mg mL^−1^.

### Vesicle preparation for supported lipid bilayer formation

1,2-Dioleoyl-*sn-glycero*-3-phosphocholine (DOPC) lipids dissolved in chloroform and bacterial lipid extracts dissolved in a 1 : 1 ratio of chloroform and methanol were mixed together at a mass ratio of 70 : 30, and the solvent mixture was evaporated by a gentle stream of nitrogen gas to form a dried thin lipid film on the wall of a glass vial. The dried film was re-dissolved in 10 mM Tris buffer solution [pH 7.5] with 50 mM NaCl at a 1 mg mL^−1^ nominal lipid concentration to form multilamellar vesicles. The vesicles were then extruded through a track-etched polycarbonate membrane with 50 nm diameter pores using an Avanti Mini-Extruder. The mean diameter of the extruded vesicles was approximately 70 nm, as verified by dynamic light scattering measurements.^23^ The vesicles were diluted to 0.2 mg mL^−1^ in 10 mM Tris buffer solution [pH 7.5] with 250 mM NaCl, with a positive osmotic pressure intended to aid the rupture of adsorbed vesicles^24,25^ for preparation of supported lipid bilayers on silica surfaces.

### Membrane binding assay by quartz crystal microbalance-dissipation (QCM-D) measurements

QCM-D experiments were conducted with a Q-Sense E4 instrument (Q-Sense AB, Gothenburg, Sweden) in order to characterize the interaction between COEs and supported lipid bilayers. The QCM-D technique monitors changes in the resonance frequency (Δ*f*) and energy dissipation (Δ*D*) of an oscillating, piezoelectric quartz crystal sensor chip as functions of time and these measurement signals are sensitive to an adsorbate's acoustic mass and viscoelastic properties, respectively.^26^ The sensor chip had a fundamental frequency of 5 MHz and a sputter-coated, 50 nm thick layer of silica (model no. QSX 303, Q-Sense AB). The experimental data was collected at the third (*n* = 3), fifth (*n* = 5), seventh (*n* = 7), ninth (*n* = 9) and eleventh (*n* = 11) odd overtones using the QSoft software program (Q-Sense AB) and the data was normalized according to the overtone number. The presented data were collected at the fifth overtone, which is representative of the other overtones. For SLB formation, the vesicle fusion method was employed and the typical lipid composition was 70 w/w% 1,2-dioleoyl-*sn-glycero*-3-phosphocholine (DOPC) and 30 w/w% *E. faecalis* lipid extract was selected following optimization experiments with 30, 50 and 80 w/w% bacterial extract as it best promoted good quality bilayer formation. Pure DOPC lipid compositions were used for control experiments as well as 70 w/w% DOPC and 30 w/w% 1-palmitoyl-2-oleoyl-*sn-glycero*-3-phospho-(1′-*rac*-glycerol) (POPG) lipid compositions. It was not possible to form a stable membrane using wild type OG1RF extracts, POPG, a representative lipid of Gram positive bacteria that is commonly used in model systems, was used instead.^27^ In the experiments, baseline QCM-D signals were stabilized using a 10 mM Tris buffer solution [pH 7.5] with 150 mM NaCl. Following stabilization, the solution was exchanged to 10 mM Tris buffer solution [pH 7.5] with 250 mM NaCl and then 0.2 mg mL^−1^ lipid vesicles in the 250 mM NaCl Tris buffer were added. After SLB formation, the sensor chip was rinsed with 250 mM NaCl Tris buffer, followed by buffer exchange with 150 mM NaCl Tris buffer.

### Model membrane permeabilization assay by electrochemical impedance spectroscopy

Gold electrodes supplied by SDx Tethered Membranes (Roseville, New South Wales, Australia) were coated with 10% benzyl-disulphide-bis-tetraethyleneglycolmonophytane tethering groups and 90% benzyl-disulphide-bis-tetraethyleneglycol spacer lipids in ethanol. The assembled TethaPlate (SDx Tethered Membranes, Roseville, NSW) contained six wells with 2.1 mm^2^ electrodes per cartridge and lipids were deposited on the sensor surface in order to prepare the tethered bilayer lipid membranes (tBLMs). Subsequent assembly of the lipid bilayer was performed by the addition of 8 μL of 3 mM of diphytanyl diether phosphatydyl choline (DPEPC) and glycerol diphytanyl ether (GPDE) at a 70 : 30 molar ratio mixed with the appropriate bacterial lipid extract at the selected SDx lipid : bacterial lipid extract mass ratio. After 2 min incubation, the flow cell was rapidly rinsed with 3 × 100 μL of 10 mM Tris buffer solution [pH 7.5] with 150 mM NaCl, resulting in spontaneous formation of the tBLM. Following tBLM formation, 100 μL of sample was injected into each well. tethaPod (SDx Tethered Membranes) and tethaPATCH (SDx Tethered Membranes) were used as the membrane conductance and capacitance reader and potentiostat connectivity unit, respectively. All measurements were collected and analyzed using tethaQUICK software (SDx Tethered Membranes), as previously described.^28,29^

## Results and discussion

### MIC of COE1-3C and COE1-3Py against *E. faecalis* OG1RF

Both COE1-3C and COE1-3Py inhibit the growth of wild type *E. faecalis* OG1RF at 2 and 1 μM, respectively (Table 1). The lower MIC of COE1-3Py may be attributed to the substitution of the trialkylammonium group of COE1-3C with the pyridinium cation.^16^

**Table 1:** Minimum inhibitory concentrations of COE1-3C, COE1-3Py and daptomycin

Strain	Description	Growth conditions	MIC		
			COE1-3C	COE1-3Py	Daptomycin
***E. faecalis*** OG1RF	Wild type	BHI	2 μM	1 μM	1.23 μM
EFC3C^a^	COE1-3C resistant	BHI	8 μM	1 μM	9.87 μM
EFC3Py^b^	COE1-3Py resistant	BHI	8 μM	16 μM	9.87 μM
DAP 21^c^	Daptomycin resistant	BHI	2 μM	1 μM	78.96 μM
DAP 22^d^	Daptomycin resistant	BHI	2 μM	1 μM	78.96 μM
***E. faecalis*** OG1RF	Wild type	BHI + palmitic acid C_16:0_ (10 μg mL^−1^)	2 μM	1 μM	ND
***E. faecalis*** OG1RF	Wild type	BHI + *cis*-vaccenic acid C_18:1 ω7c_ (10 μg mL^−1^)	8 μM	2 μM	ND
***E. faecalis*** OG1RF	Wild type	BHI + *cis*-9,10-methyleneoctadecanoic acid C_19:0 cyclo ω8c_ (12.5 μg mL^−1^)	8 μM	4 μM	ND

a COE1-3C resistant strains with in frame deletion in *liaF* at position 179 and a substitution in the intergenic region between *treB* (PTS family trehalose porter, IIBC component) and *gloA6* (lactoylglutathione lyase).

b COE1-3Py resistant strains with a non-synonymous substitution at position 97 in the *liaR* gene.

c DAP21 strains have mutations in cardiolipin synthase 1 (cls1; RF10364), a gene encoding a putative chaperone protein regulated by the *liaFSR* operon (RF11464) and hypothetical membrane protein (RF11507).^21^

d DAP22 strains have mutations in gene encoding a putative chaperone protein regulated by the *liaFSR* operon (RF11464) and hypothetical membrane protein (RF11507) along with mutations in cardiolipin synthase 2 (cls2; RF11324) and a putative metal-dependent HD-domain-containing hydrolase (RF11901).^21^ ND – not determined.

### Resistance of *E. faecalis*, EFC3C and, EFC3Py to COE1-3C and COE1-3Py

By serial passage of *E. faecalis* in incrementally increasing concentrations of COEs, it was possible to produce mutants that were moderately resistant to COE1-3C (EFC3C, MIC = 8 μM) and COE1-3Py (EFC3Py, MIC = 16 μM), achieving a 4 and 16 fold increase in MIC, respectively (Table 1). EFC3C was not cross resistant to COE1-3Py but EFC3Py was cross resistant to COE1-3C. This suggests a different mechanism of action between the two COEs used here, caused by differences in their molecular structure, suggesting either an affinity for different lipid classes or through some nuanced interaction with the acyl chains in the lipid membrane. Understanding such interactions is of scientific importance and is critical for the rational development and redesign of antimicrobial compounds. The 4 and 16 fold increase in MIC between the wild type and the strains adapted to COE1-3C and COE1-3Py, respectively, is relatively small compared to other compounds. For example, for *in vitro* evolved daptomycin resistant *E. faecalis* strains, a 256–512 fold increase in MIC compared to the sensitive strain was reported.^21,30^

Previous *in vitro* evolution studies with daptomycin (DAP) and resistant variants of *E. faecalis* V583 showed a “step pattern” of increase in DAP resistance over a 15 d period. This pattern was characterized by some of the isolates exhibiting daptomycin resistance of 128 μg mL^−1^ until day 11, 256 μg mL^−1^ until day 13, and quickly reaching peak in daptomycin resistance of 512 μg mL^−1^ on the day 14.^30^ In our study, the MIC of COEs plateaued on day 5 and day 12 for COE1-3C and COE1-3Py, respectively (Fig. 1), further changes in COE tolerance were not observed during subsequent passaging in the presence of COE for the remaining three or ten day periods. The inability of *E. faecalis* to achieve further increase in resistance to COE is an interesting and desirable feature of this class of compounds.

### EFC3C and EFC3Py are 8-fold more resistant to daptomycin than wild type

Daptomycin is a last resort antibiotic for complex enterococcal infections and whose mechanism of action involves a lipid based interaction with Gram positive bacteria such as *Bacillus subtilis* and *E. faecalis*.^19,20,31,32^ It behaves as a cationic antimicrobial peptide (cAMP) in the presence of calcium ions.^33,34^ The suspected lipid based interaction of COEs and their membrane perturbation properties share similarities to cAMPs^16,17^ appear superficially similar to the mechanism of action of daptomycin. For this reason, we studied the cross resistance of EFC3C and EFC3Py to daptomycin. In our study, EFC3C and EFC3Py were mildly resistant to daptomycin at a concentration of 9.87 μM, which is around 8 fold higher than the wild type (MIC = 1.23 μM) (Table 1).

### The genetic basis for EFC3C and EFC3Py resistance differ

To confirm that the observed differences in the MIC of COE1-3C and COE1-3Py elicited a unique resistance in the adapted EFC3C and EF3CPy strains, whole genome sequencing was performed. The genomes of EFC3C and EFC3Py harbored mutations associated within LiaFSR, a three component cell envelope homeostasis and stress response system. LiaFSR is conserved in Gram positive bacteria and functions as a damage sensing and signal transducing system.^35^ It includes a transmembrane histidine kinase (LiaS), cytosolic response regulator (LiaR) and a membrane bound negative regulator (LiaF). LiaF interacts with LiaS to maintain the LiaFSR regulon in an “OFF” state. In the presence of a membrane stress, LiaS responds by phosphorylating LiaR, which in turn leads to the transcription of operons with LiaR binding regions.^36^ The LiaFSR homologous systems involved in cell envelope associated stress response are widespread in the *Firmicutes* except in *Lactobacillus* and *Clostridium*.^37–41^ Sequencing of the EFC3C strain revealed an in frame deletion of an isoleucine codon at position 179 in *liaF*, a negative regulator of LiaR in the LiaFSR system as well as a single nucleotide substitution in the intergenic region of *treB* (PTS family trehalose porter, IIBC component) and *gloA6* (lactoylglutathione lyase) (Table 3). In contrast, a non-synonymous substitution at position 97 was found in *liaR* for EFC3Py. Additional SNPs were also identified in EFC3Py at gene loci: OG1RF_11256, OG1RF_11765 and OG1RF_11767, respectively. Sequencing of strains collected during the course of the *in vitro* passaging revealed that these strains harbored mutations exclusively in these loci prior to the accumulation of *liaR* associated mutation. The MICs of COEs to these isolates were identical to the wild type *E. faecalis* and hence it was concluded that these mutations did not play a critical role in resistance to COE1-3C and COE1-3Py.

**Table 2:** Percentage of total membrane content as determined by fatty acid analysis

Fatty acid	% of total membrane content^a^		
	*E. faecalis* OG1RF wild type	EFC3C	EFC3Py
C_10:0_	0.00 ± 0.00	0.05 ± 0.05	0.02 ± 0.04
C_12:0_	0.29 ± 0.24	0.19 ± 0.25	0.20 ± 0.28
C_14:0_	3.55 ± 0.27	3.61 ± 0.46	3.46 ± 0.42
C_15:1 ω8c_	0.06 ± 0.12	0.00 ± 0.00	0.07 ± 0.10
C_16:0_	40.07 ± 1.03	37.73 ± 1.55*	36.78 ± 0.66*
C_17:0 cyclo_	ND	0.10 ± 0.21	0.04 ± 0.09
C_17:1 ω8c_	1.24 ± 1.55	0.86 ± 1.71	0.00 ± 0.00
C_18:1 ω7c_	33.85 ± 0.88	31.93 ± 1.61*	35.93 ± 1.43*
C_18:0_	5.65 ± 0.70	5.26 ± 0.43	5.45 ± 0.32
C_18:1 ω9c_	0.07 ± 0.13	0.00 ± 0.00	0.00 ± 0.00
C_19:1*iso I*_	0.44 ± 0.06	0.41 ± 0.05	0.36 ± 0.06
C_19:0 cyclo ω8c_	7.37 ± 0.56	12 ± 0.69*	10.18 ± 0.37*
C_19:0 10 methyl_	0.82 ± 0.07	1.31 ± 0.28	0.98 ± 0.20
C_20:2 ω6,9c_	0.00 ± 0.00	0.05 ± 0.10	0.00 ± 0.00
C_20:1 ω7c_	0.40 ± 0.08	0.51 ± 0.06	0.59 ± 0.10
C_16:1 ω7c/15 *iso* 2OH_	3.92 ± 0.30	4.07 ± 0.26	4.00 ± 0.25
C_15:0 *iso* 2OH/16:1ω7c_	1.09 ± 0.08	1.10 ± 0.08	1.11 ± 0.05
C_17:1 *iso*/*antei* B_	0.83 ± 0.11	0.68 ± 0.06	0.62 ± 0.05
C_uneluted peak_^±^	0.31 ± 0.08	0.27 ± 0.09	0.20 ± 0.13

a The values are shown as averages ± standard deviations of five different replicates of *E. faecalis* OG1RF, EFC3C and EFC3Py. Significant differences between the mutants EFC3C and EFC3Py in comparison to the wild type was calculated using two-way ANOVA and Tukey's multiple comparisons test-and corrected *p*-values indicated with *p ≤ 0.0001. ±Unknown peak eluted between C_14:0_ and C_16:1 ω7c/15 *iso* 2OH_.

**Table 3:** Single nucleotide polymorphisms in COE1-3C and COE1-3Py resistant strains EFC3C and EFC3Py

Gene locus	Description	Strain	
		EFC3C	EFC3Py
OG1RF_12213	Membrane protein (*liaF*)	Ile179 (deletion)	—
OG1RF_11256	Site-specific tyrosine recombinase XerD	—	Ala233Val (substitution)
OG1RF_11765	MerR family transcriptional regulator	—	Val134_Asp135insValVal (insertion)
OG1RF_11767	Multidrug ABC transporter ATP-binding protein	—	Thr188Asn (substitution)
OG1RF_12211	DNA-binding response regulator (*liaR*)	—	Ala98Val (substitution)

The MIC of COEs against daptomycin resistant strains DAP 21 and DAP 22 (Table 1), which were also evolved from *E. faecalis* OG1RF, was determined.^21^ We observed that the highly daptomycin resistant strains (MIC = 78.96 μM) did not show increased resistance to either of the COEs, with MICs of 2 and 1 μM for COE1-3C and COE1-3CPy, respectively. This suggests the mechanisms of resistance for the two classes of compounds are somewhat different, although there is some role of LiaFSR in mediating low levels of daptomycin resistance as well as resistance to the COEs. While the precise role of LiaFSR in daptomycin resistance is unknown; it is hypothesized that this effect is mediated as a consequence of changes associated with LiaFSR function. The different mechanism between the two also provides the first evidence that COEs are inhibitory to drug resistant strains.

### Fatty acid profiles of EFC3C, EFC3Py and *E. faecalis* wild type

The membrane fatty acid profiles of EFC3C and EFC3Py strains along with the wild type were determined using FAME analysis. The membranes of stationary phase cells of *E. faecalis* OG1RF are primarily composed of palmitic acid (C_16:0_), *cis*-vaccenic acid (C_18:1 ω7c_) and *cis*-9,10-methyleneoctadecanoic acid (C_19:0 cycloω8c_, a cyclopropane fatty acid).^42,43^ In the COE adapted mutants, the same dominant fatty acids were observed, although their relative contributions were significantly different from the wild type *E. faecalis*.

**Fig. 2:**
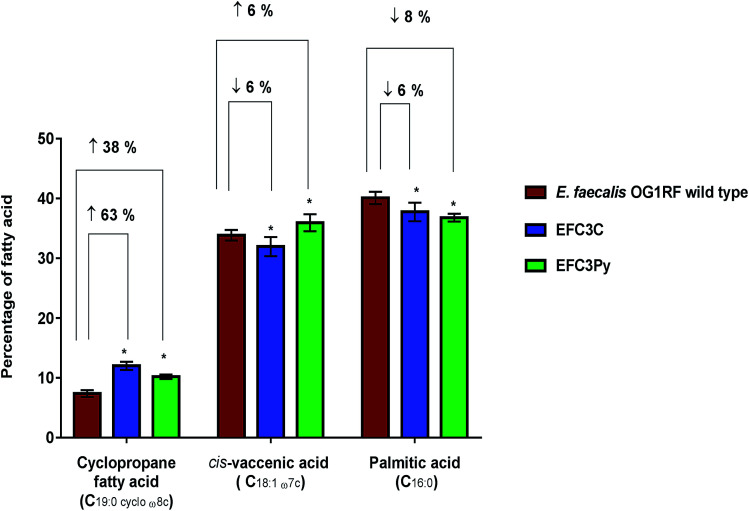
Percentage changes in the three dominant fatty acids (>10% each of the total fatty acid content) determined as a percentage of total membrane content; shown as averages ± standard deviations of five biological replicates of *E. faecalis* OG1RF wild type, EFC3C and EFC3Py. Significant differences between the mutants EFC3C and EFC3Py compared to the wild type was calculated using two-way ANOVA and Tukey's multiple comparisons test and corrected *p*-values indicated with *p ≤ 0.0001.

In both EFC3C and EF3CPy, the level of cyclopropane fatty acid (C_19:0 cycloω8c_) in the membrane, increased by 63% and 38%, respectively (*p* ≤ 0.001, *n* = 5), in comparison to the wild type. Fatty acid analysis also revealed a 6 and 8% decrease in palmitic acid (C_16:0_) (*p* ≤ 0.001 for EFC3C and EFC3Py, *n* = 5) (Table 2, Fig. 2) when compared with the wild type. Additionally, for EFC3C we observed a 6% decrease (*p* ≤ 0.001, *n* = 5) in *cis*-vaccenic acid (C_18:1 ω7c_) while for EFC3Py there was an increase of 6% (*p* ≤ 0.001, *n* = 5). The data suggest that cyclopropane fatty acids, are involved in COE resistance in general (and hence helps explain the cross resistance between COE1-3C and COE-3Py) but that increased *cis*-vaccenic acid was specifically associated with COE1-3Py resistance. Cyclopropane fatty acids are implicated in membrane stress and it is thought that they improve the stability of the membrane under extreme conditions, such as high osmotic pressure and high temperature, while also reducing the permeability to toxic compounds.^44–47^

### Effect of fatty acid supplementation to *E. faecalis* on sensitivity to COEs

Whilst statistically significant changes in fatty acid composition were associated with membrane adaptations, it is unclear if these fatty acids are responsible for COE resistance in *E. faecalis*. *E. faecalis* OG1RF does not possess genes for β-oxidation as observed by homology searches with the existing genomic sequences of *E. faecalis*.^48^ Therefore, *E. faecalis* cannot degrade fatty acids from the medium but can incorporate exogenous fatty acids into its membrane.^43,48^ Manipulating the fatty acid content of the growth medium is an effective way to influence membrane composition.^43,48^*E. faecalis* cultures were grown in the presence of different fatty acids to determine if changes in the membrane fatty acid composition could alter the MIC of COE1-3C or COE1-3Py. Supplementation of palmitic acid into the growth medium did not increase the susceptibility of *E. faecalis* to either COE1-3C or COE1-3Py (Table 1). This observation is in line with the FAME analysis data, which showed a decrease in palmitic acid, suggesting that supplementation of this fatty acid would not influence resistance against COEs. Supplementation with *cis*-vaccenic acid increased the MIC of COE1-3C and COE1-3Py in the wild type by two fold and four fold, respectively (Table 1). This was in agreement with the fatty acid measurements of EFC3Py which suggests a positive correlation between *cis*-vaccenic acid with tolerance to higher concentrations of COE (Fig. 2). In contrast, the MIC of COE1-3C against wild type strain increased in the presence of *cis*-vaccenic acid and possibly explains the resistance of EFC3Py to COE1-3C (Table 1).

In this, study, the addition of *cis*-9,10-methyleneoctadecanoic acid decreased the sensitivity of wild type *E. faecalis* as evident by an increase in MIC (Table 1). This shows that cyclopropane fatty acid levels also affect COE resistance.

Recently, it has been shown that supplementation of exogenous fatty acids into the membrane of *E. faecalis* serves to protect the cell membrane from daptomycin.^43,48^ These results suggest that subtle changes to membrane fatty acid compositions are associated with daptomycin and COE resistance but do not fully explain them. In its entirety, COE resistance is likely to include, lipid and protein components.

### Physical aspects of COE resistance

*cis*-Vaccenic acid and cyclopropane fatty acids are likely to alter the physical and chemical properties of the membrane.^43–45,49^ One possible method whereby fatty acid changes could increase resistance to COEs is by inhibiting their insertion into the membrane. Alternatively, reordering of the existing membrane fatty acids could limit the extent to which COE interactions disrupt membrane properties such as permeability.

**Fig. 3:**
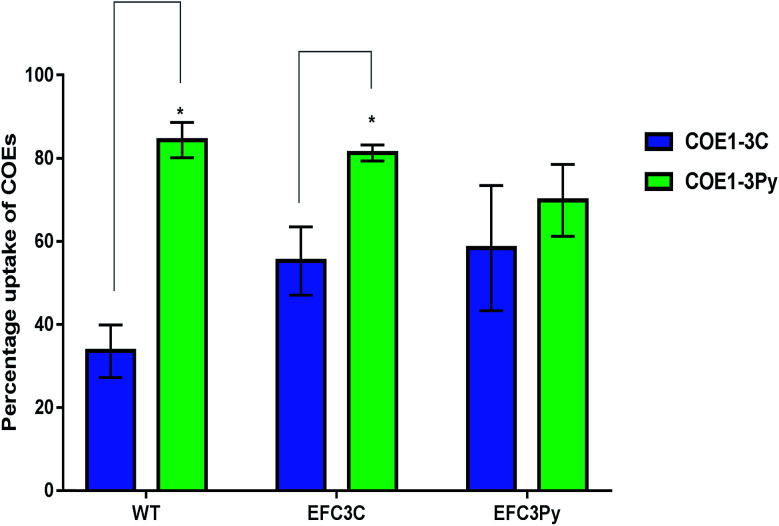
The relative uptake of 5 μM COEs in *E. faecalis* OG1RF wild type (WT), EFC3C and EFC3Py determined by measuring the absorbance at the wavelength of maximum absorbance of a 5 μM solution of COEs. Significant differences between the treatments in the respective strains was calculated by *t*-test and *p*-values indicated with *p ≤ 0.01.

COE1-3Py interacts more readily with the membrane than COE1-3C (Fig. 3). However, the mutation in EFC3C appeared to preferentially increase uptake of COEs as compared to the wild-type strain as demonstrated by the accumulation of COE1-3C in the wild type (≈34%) compared to EFC3C (≈55%). Evidence for this is illustrated by the fact that there is no significant difference between the relative uptake of the two COEs in EFC3Py but that this difference is significant (*p* ≤ 0.01) in both the wild type and EFC3C (Fig. 3). Additionally, the magnitude of COE1-3Py uptake is less (≈70%) in EFC3Py than it is in the wild type (≈84%).

Interfacial self-assembly of π-conjugated polymers into fibrils and aggregates is known to occur and could impact uptake and toxicity.^50^ If self-assembly occurs significantly outside the cell, it would be expected to mitigate toxicity by hindering uptake whereas assembly driven by hydrophobic interactions in the bilayer could increase membrane perturbation.^3^ The degree of COE uptake observed suggest that interfacial self-assembly is not driving membrane perturbation.

The observations presented in Fig. 3, suggest that the adaptive response on exposure to COE1-3Py, which accumulates more readily in the membrane, would be to exclude it to preserve membrane integrity. EFC3C, however, appears to mediate adaptation through COE accumulation, suggesting that the particular fatty acid changes promote accommodation of COE in the membrane. Cyclopropane fatty acids have been show to simultaneously promote acyl chain order and membrane fluidity, a property which is consistent with the accommodation of the COE in the membrane.^44^ The increased ordering that would be expected to occur from an increase in the cyclopropane fatty acid, *cis*-9,10-methyleneoctadecanoic acid (C_19:0 cyclo ω8c_), which is accumulated to the highest degree in EFC3C, also supports this finding.

Such mechanistic insights into the precise effects of single membrane components cannot be achieved in live cells, but could be studied in model systems with precise compositions of purified bacterial lipids with the exact acyl chains postulated to be important drivers of the phenomenon of interest, the development of such model systems should be the focus of future research efforts.

**Fig. 4:**
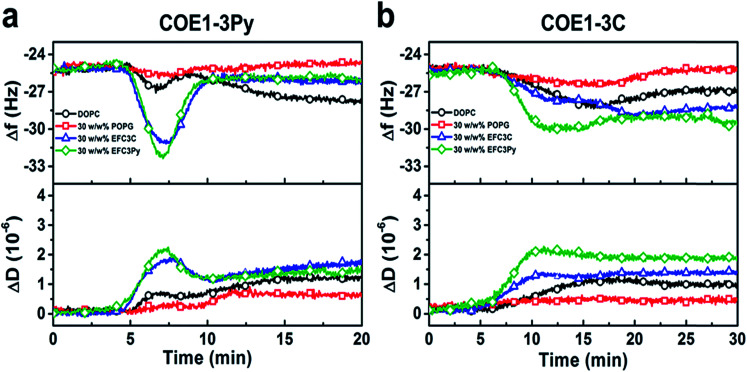
QCM-D monitoring of COE interactions with supported lipid bilayers. Resonance frequency (Δ*f*) and energy dissipation (Δ*D*) signals are presented as functions of time. The baseline signals correspond to fabricated SLBs on silica-coated sensor chips, and 5 μM COE compounds were added starting at *t* = 5 min under continuous flow conditions. All measurement shifts are relative to equivalent buffer conditions.

To investigate the physicochemical interactions driving the antibacterial activity of the COE compounds against bacterial cell membranes, quartz crystal microbalance-dissipation (QCM-D) experiments were conducted to evaluate membrane binding to supported lipid bilayers (SLBs) containing defined model phospholipids or more complex mixtures of bacterial lipid extracts. The QCM-D results demonstrated that both COE1-3C and COE1-3Py exhibited preferential binding to bacterial lipid SLBs over the model phospholipid compositions, as indicated by larger frequency shifts which are proportional to the amount of adsorbed mass as well as larger energy dissipation shifts suggestive of more extensive morphological perturbations with increased viscoelastic properties (Fig. 4).^51^ Interestingly, the membrane binding kinetics was quite different for the two compounds (Fig. 4). COE1-3C association with the bacterial lipid SLBs exhibited monotonic adsorption kinetics, whereas COE1-3Py had more complex, two-step interaction kinetics, supporting that the interaction involves some degree of membrane perturbation, with associated changes in the SLB's viscoelastic properties.^25^ This observation agrees well with the increased hydrophobicity of COE1-3Py, which is the main driving force for COE uptake.^52,53^ Additionally, the two compounds showed minimal interactions with SLBs composed of the model cell-membrane mimicking phosphatidylcholine (DOPC) lipids alone, which is representative of mammalian cells, providing first insights that COE binding shows some degree of selectivity for bacterial membrane lipid components. These data support the spectroscopic observations obtained from living systems.

**Fig. 5:**
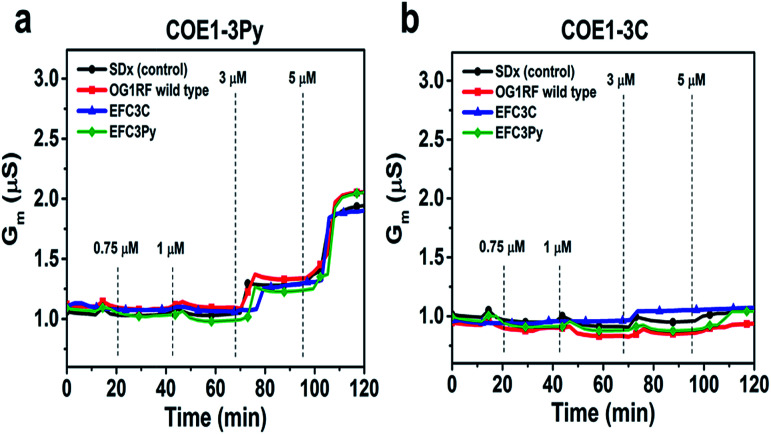
Electrochemical impedance spectroscopy (EIS) measurements using a tethered lipid bilayer membrane platform. Increasing concentrations of compound were added at the indicated time point. Control is standard tBLM lipid composition without bacterial cell lipid extract.

To further assess the effect of COE binding on membrane permeability, we conducted electrochemical impedance spectroscopy (EIS) measurements using a tethered lipid bilayer membrane platform. In direct agreement with the QCM-D measurements and the adsorption studies, addition of COE1-3Py caused concentration-dependent membrane leakage by pore formation or lysis, as evidenced by increased conductivity across the model membranes (Fig. 5). A marked transition in membrane permeabilization occurred around 1–3 μM. In contrast, addition of COE1-3C led to markedly reduced measurement responses in the EIS experiments, which is also consistent with the monotonic binding observed in the QCM-D measurements and indicates a lower degree of membrane permeability. Of particular interest, membrane extracts from mutants that are resistant to COEs showed a modified, molecule-specific response to COE treatment. For example, extracts from the EFC3Py strain exhibited increased porosity upon treatment with COE1-3Py but the response to COE1-3C treatment was negligible. Thus, there was a specific membrane response to each of the COEs and this is supported by the genetic and fatty acid data (Table 1 and Fig. 2).

## Conclusions

It has been shown that two structurally related compounds, COE1-3C and COE1-3Py, which differ only in the presence of a terminal pyridinium group (Fig. 1) have different membrane-perturbing effects on *E. faecalis* and that this is apparent from the different genetic and fatty acid adaptations to each of these COEs. Furthermore, significant resistance to these compounds was not developed in *E. faecalis*. These findings are of significance from a molecular design point of view as they confirm that minor structural modifications can lead to very different antimicrobial effects. These findings show that fatty acid changes play a significant role in the physical basis of COE resistance. It is possible that other factors also contribute to this resistance and hence, future work should address the roles of changes in protein and gene expression in response to COE exposure to fully explain the basis of COE resistance. Further work to understand such interactions in fine detail will lead to the design and synthesis of a new class of potent antimicrobials which may not elicit drug resistance whilst also avoiding toxicity to the host by increasing the specificity for the microbial membranes.

## Conflicts of interest

There are no conflicts of interest to declare.

## Supplementary Material
